# Strategies to Improve the Efficacy of Dendritic Cell-Based Immunotherapy for Melanoma

**DOI:** 10.3389/fimmu.2017.01594

**Published:** 2017-11-20

**Authors:** Kristian M. Hargadon

**Affiliations:** ^1^Hargadon Laboratory, Department of Biology, Hampden-Sydney College, Hampden-Sydney, VA, United States

**Keywords:** dendritic cell, tumor, cancer immunotherapy, melanoma, immune suppression, immunogenic cell death, immunometabolism, microbiome

## Abstract

Melanoma is a highly aggressive form of skin cancer that frequently metastasizes to vital organs, where it is often difficult to treat with traditional therapies such as surgery and radiation. In such cases of metastatic disease, immunotherapy has emerged in recent years as an exciting treatment option for melanoma patients. Despite unprecedented successes with immune therapy in the clinic, many patients still experience disease relapse, and others fail to respond at all, thus highlighting the need to better understand factors that influence the efficacy of antitumor immune responses. At the heart of antitumor immunity are dendritic cells (DCs), an innate population of cells that function as critical regulators of immune tolerance and activation. As such, DCs have the potential to serve as important targets and delivery agents of cancer immunotherapies. Even immunotherapies that do not directly target or employ DCs, such as checkpoint blockade therapy and adoptive cell transfer therapy, are likely to rely on DCs that shape the quality of therapy-associated antitumor immunity. Therefore, understanding factors that regulate the function of tumor-associated DCs is critical for optimizing both current and future immunotherapeutic strategies for treating melanoma. To this end, this review focuses on advances in our understanding of DC function in the context of melanoma, with particular emphasis on (1) the role of immunogenic cell death in eliciting tumor-associated DC activation, (2) immunosuppression of DC function by melanoma-associated factors in the tumor microenvironment, (3) metabolic constraints on the activation of tumor-associated DCs, and (4) the role of the microbiome in shaping the immunogenicity of DCs and the overall quality of anti-melanoma immune responses they mediate. Additionally, this review highlights novel DC-based immunotherapies for melanoma that are emerging from recent progress in each of these areas of investigation, and it discusses current issues and questions that will need to be addressed in future studies aimed at optimizing the function of melanoma-associated DCs and the antitumor immune responses they direct against this cancer.

## Introduction

Melanoma is responsible for ~10,000 deaths in the United States and ~55,000 deaths worldwide each year, making it the cause of over 75% of skin cancer-related deaths (1, 2). Importantly, data collected by the SEER Program show that melanoma incidence rates have continually risen the last 40 years (3), and a recent study projects melanoma incidence to continue increasing through at least 2022 (4). In the U.S. alone, annual costs for treatment and productivity losses associated with melanoma are near $3.3 billion (5). These numbers are even more staggering when considering the U.S. ranks only third in melanoma incidence worldwide (6), thus highlighting the need to address melanoma as a global public health concern.

Although it is the least common form of skin cancer, melanoma is by far the most lethal due to its propensity to metastasize to several vital organs, including the brain, lungs, liver, and other visceral organs (7). While surgical removal of primary melanomas is highly successful in eradicating disease prior to metastasis, many melanoma patients are not diagnosed until later stages of malignant disease. In these cases, surgery is often not possible or is largely ineffective (8). Moreover, traditional therapies such as chemotherapy and radiation also exhibit limited efficacy against malignant melanoma and are characterized by variable response rates, lack of durable responses, toxicity, and minimal impact on survival (9, 10). In recent years, important insights into the basic biology of melanoma progression have led to the development of several targeted therapies that have shown promise in the treatment of metastatic melanoma patients. In particular, vemurafenib, trametinib, dabrafenib, and other inhibitors of the BRAF–MEK signaling pathway that is hyperactive in melanoma patients bearing BRAF^V600^ mutations have proven superior to traditional chemotherapy in terms of both antitumor activity and clinical outcome (11–13). Unfortunately, drug resistance to BRAF or MEK inhibitors often develops within the first year of treatment and is accompanied by disease progression in many melanoma patients (14–16). While combination therapy with BRAF–MEK inhibitors delays melanoma progression and improves overall survival as compared to monotherapy, development of multi-drug resistance still leads to disease relapse in many patients (17, 18). A similar story has unfolded with regard to even the most promising immunotherapies for melanoma. Checkpoint blockade therapies with monoclonal antibodies targeting inhibitory receptors such as CTLA-4 and PD-1 on CD8^+^ T lymphocytes have been developed to override cell intrinsic mechanisms that limit overstimulation of T cells and have dramatically improved both antitumor T cell function and clinical responses in melanoma patients. Both monotherapy and combinatorial approaches with nivolumab (anti-PD-1), pembrolizumab (anti-PD-1), and ipilimumab (anti-CTLA-4) have been promising, with reports of complete and objective responses in as high as 22 and 61% of melanoma patients, respectively (19–26). Despite these successes, though, many melanoma patients do not respond to these therapies, and others often experience disease relapse in as early as the first few months of treatment (27–29). Likewise, adoptive cell transfer (ACT) therapies that employ either naturally occurring tumor-infiltrating lymphocytes or genetically engineered T lymphocytes have produced complete tumor regression in as high as 25% of melanoma patients, though many other patients receive no clinical benefit from these regimens (30, 31). Therefore, while recent advances in the treatment of metastatic melanoma are encouraging, it is critical that we continue to explore strategies that will expand treatment options and optimize clinical outcome for patients with this disease.

Dendritic cells (DCs) have long been appreciated for their roles in the induction and maintenance of antitumor immune responses and are known to be critical regulators of both antitumor immune activation and immune tolerance. This dichotomy is highlighted by the variable outcomes of early trials employing DC-based therapies in melanoma patients. While tumor vaccines targeting host antigen (Ag)-presenting cells *in situ* or utilizing exogenous tumor Ag-loaded DC induced immunogenic responses that correlated with clinical benefits in a modest percentage of patients (32–35), many patients exhibited no clinical response to these therapies, and some immunization maneuvers even led to diminished tumor-specific T cell responses and the induction of immune tolerance, thereby potentially exacerbating disease progression (36, 37). Lessons learned from these first-generation cancer vaccines guided second-generation vaccination strategies that aimed to improve upon previous failures by (1) targeting tumor Ag to particular DC subsets *in situ* or (2) employing maturation cocktails to promote the immunostimulatory activity of exogenously generated monocyte-derived DCs. In addition to pulsing these latter DCs with recombinant synthetic peptides or tumor cell lysates, other approaches for tumor Ag loading onto exogenous DCs were also explored, including RNA/DNA electroporation and fusion of tumor cells to DCs. Details of these approaches have been described more extensively in recent reviews (38–40), and their translation to the clinic is highlighted in a recent Trial Watch (41). In brief, despite the improved immunogenicity of many of these approaches, they have unfortunately not been met with the success of checkpoint blockade and ACT therapies, and objective response rates have rarely exceeded 15%. Nevertheless, significant efforts in recent years have further improved our understanding of factors that regulate DC function in the context of cancer, and insights from this work have suggested novel strategies for improving the immunogenicity of both endogenous and exogenous DC. At the same time, advances in genetic engineering and other approaches that enable the manipulation of DC function are spearheading the translation of this basic research on DC immunobiology into novel clinical applications. Together, these findings have reinvigorated the pursuit of cutting-edge approaches that take advantage of the potential of DC as potent stimulators of robust, targeted antitumor immune responses, offering great promise for the future of DC-based cancer immunotherapies.

## Next-Generation DC-Based Immunotherapy for Melanoma

Although first- and second-generation DC vaccines, as well as other tumor Ag-based vaccines, have not yielded significant clinical benefit in a large percentage of melanoma patients to date, their relatively good safety profiles and ability to induce antitumor immune responses in some patients have encouraged the pursuit of next-generation melanoma vaccines that aim to improve upon the previous limitations of DC-based immunotherapy for this cancer. A major focus of one class of next-generation DC vaccines is the utilization of naturally occurring DC subsets, which differs from the artificial *ex vivo* generation of monocyte-derived and CD34^+^ precursor-derived DC that predominated both first- and second-generation DC vaccination protocols. Though large clinical trials are needed to define which DC subsets provide optimal therapeutic efficacy in particular settings, early trials with plasmacytoid DC (pDC) and CD1c^+^ myeloid DC (mDC) have both shown promise in melanoma patients. Intranodal injection of pDC that had been activated and pulsed with melanocyte differentiation Ag-derived peptides into tumor-free lymph nodes of patients with distant metastatic melanoma-induced Ag-specific CD8^+^ T cell responses in nearly 50% of patients, and although the sample size was too small to make definitive assessments of clinical efficacy, a comparison of clinical outcomes for these patients versus matched control patients undergoing dacarbazine chemotherapy suggest vaccination benefits for both progression-free survival and overall survival (42). Likewise, immunization of stage IIIc/IV melanoma patients with autologous, peptide-pulsed CD1c^+^ mDC promoted Ag-specific CD8^+^ T cell responses in 33% of tested patients and induced long-term progression-free survival (12–35 months) in nearly 30% of patients (43). Other next-generation vaccination approaches currently being explored include immunization with tumor-specific neoantigens (either alone or loaded onto DC) that promote responses against mutated tumor-specific epitopes (44–46) as well as maneuvers that induce local or systemic activation of endogenous, tumor Ag-presenting DC (47, 48). These next-generation DC-based vaccines and the ways in which they might be incorporated as part of combinatorial regimens into the current cancer immunotherapy landscape that is being dominated by checkpoint blockade and ACT therapies have recently been reviewed more thoroughly elsewhere (49). Importantly, optimization of these next-generation approaches going forward will require careful consideration of the many factors that have emerged as regulators of DC function in the context of cancer. In this regard, this review highlights recent advances in our understanding of factors that influence DC function in melanoma immunity, including the immunogenicity of tumor cell death, immunosuppressive networks within the tumor microenvironment, tumor-altered immunometabolism, and microbiome-associated regulation of DC function and DC-mediated antitumor immunity. Additionally, particular focus is given to therapeutic strategies building on this knowledge that aim to improve the quality of next-generation DC-based immunotherapies for the treatment of melanoma.

## Induction of Immunogenic Cell Death (ICD) as a Means of Promoting DC-Mediated Antitumor Immunity

### ICD and DC Activation

**Figure 1:**
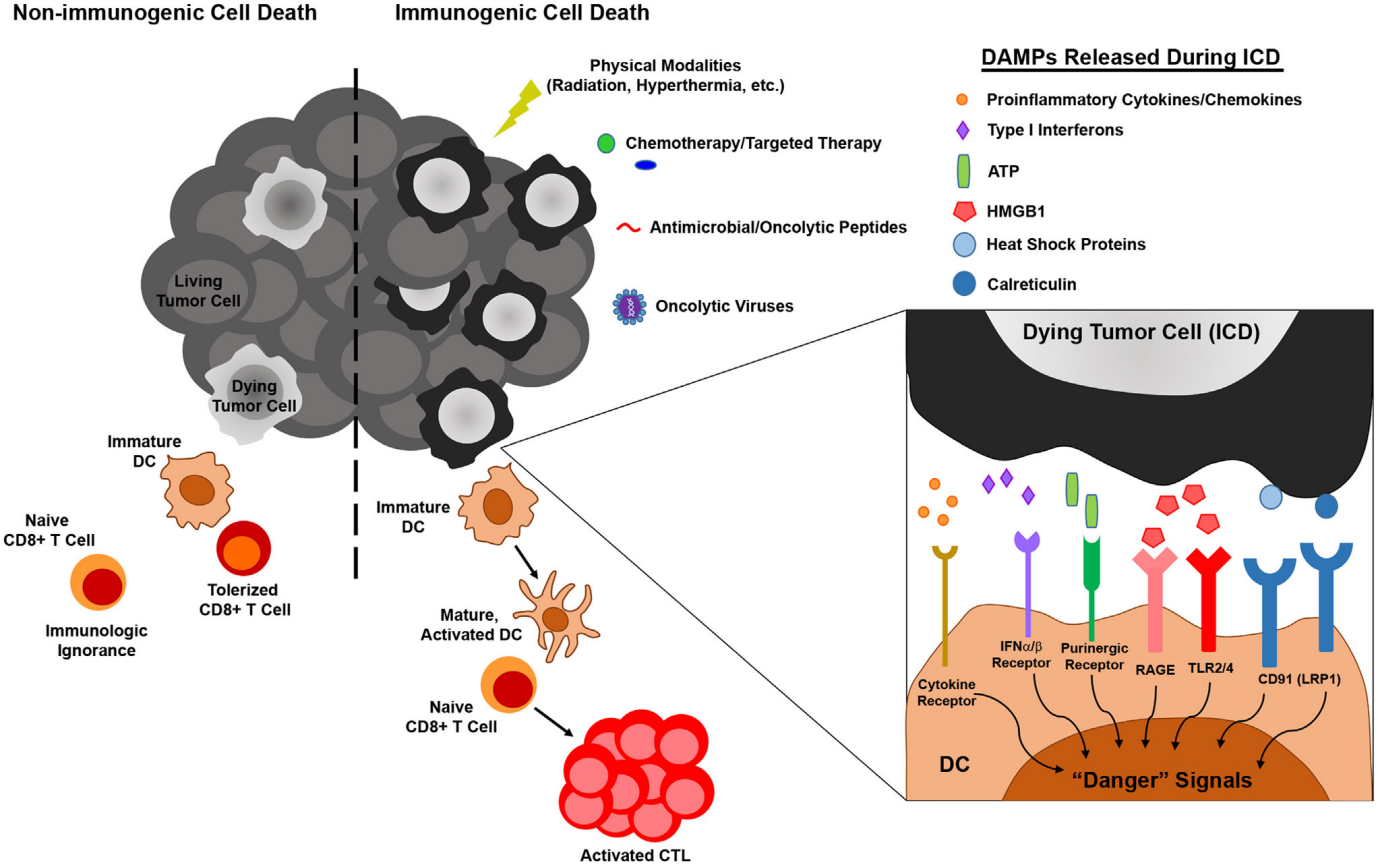
The influence of immunogenic versus non-immunogenic tumor cell death on dendritic cell (DC) maturation/activation and DC-mediated antitumor immunity. Non-immunogenic tumor cell death does not elicit DC maturation or activation, leaving DC in an immature state in which they either (1) fail to “sense” tumor cell death and therefore do not acquire tumor antigen (Ag) for presentation to naïve T lymphocytes or (2) acquire tumor Ag through phagocytosis and induce T cell tolerance. On the other hand, immunogenic tumor cell death, which can be elicited by various physical, chemical, and biological modalities, results in the release of damage-associated molecular patterns (DAMPs) that are recognized by pattern recognition receptors on DC, resulting in the delivery of “danger” signals that promote the maturation and activation of DC capable of stimulating antitumor T cell activation.

As one of the primary mediators of immune surveillance, DC function as key sentinels that aim to maintain homeostasis within the body, invoking immune tolerance in the steady state and immune activation in times of stress, such as that which occurs during a pathogenic infection. In the steady state, DCs exist as immature, inactivated cells that are highly phagocytic but tolerogenic in nature, expressing low levels of the costimulatory molecules and proinflammatory cytokines/chemokines necessary to invoke immune activation and effector cell recruitment to peripheral tissues. On the other hand, upregulation of these cell surface and soluble immunostimulatory molecules during DC maturation and activation promotes the induction of adaptive immunity capable of eliminating a particular source of Ag (50). While it was originally thought that DC maturation and activation status, and in turn the ability of DC to induce immune tolerance versus activation, was dictated solely by self/non-self discrimination (51), more recently, it has become appreciated that regardless of how self or foreign a source of Ag is, it is the microenvironmental cues within host tissues that are critical in driving the “friend or foe” decision made by DC upon Ag encounter (52). In this way, immature DC that encounter and phagocytose cells dying naturally from normal turnover can remove this cellular debris without risking aberrant autoimmune activation, while those that encounter cells dying from infection or other forms of stress (such as those ultimately imposed on at least some of the cancer cells within a growing tumor) receive “danger signals” that promote their maturation, activation, and ability to stimulate immune responses to combat the source of “danger.” In the context of cancer, several of these “danger signals” have now been identified as damage-associated molecular patterns (DAMPs) (53). These include cell surface calreticulin and other endoplasmic reticulum (ER) chaperones exposed following the unfolded protein response, autophagy-mediated or conventional secretion of ATP, interleukin-1β (IL-1β) secretion as a result of inflammasome signaling, release of high-mobility group box 1 (HMGB1), and cell surface exposure/release of annexin A1, though this latter protein has been shown to promote both DC activation (54) and inhibition (55) in different settings, and its role as a DAMP is controversial. Nucleic acids released from dying tumor cells are another well-characterized DAMP that may signal through cytoplasmic sensors such as RIG-I or the TLR7/8/9-MyD88 pathway to stimulate DC. Additionally, their induction of type I IFN secretion by dying tumor cells can also lead to autocrine signals that trigger release of chemokines such as CXCL10 that promote recruitment of immune cell populations to the tumor (53, 56). Ultimately, it is the engagement of these types of DAMPs by pattern recognition receptors on DC that “alerts” these cells to an ICD and in turn promotes their stimulation of immune reactivity against “dangerous” immunogens (Figure 1). With this revised understanding of “danger/no danger” discrimination as the key regulator of immune activation, inducers of ICD in cancer have become a major area of investigation because of their potential to promote DC-mediated antitumor immunity.

### Chemotherapy-Driven ICD and Its Potential for Activation of Endogenous Tumor-Associated DC

In recent years, a number of anticancer regimens have been investigated for their ability to induce ICD and enhance DC-based cancer immunotherapies (57–61). Interestingly, while it was once thought to be at odds with cancer immunotherapy because of its non-specific targeting of rapidly dividing cells (which could include not only tumor cells but also lymphocytes engaged in an antitumor immune response), chemotherapy has recently been revisited as a means of promoting ICD of tumor cells. Indeed, a number of chemotherapeutic agents approved for the treatment of various cancers, including doxorubicin, oxaliplatin, mitoxantrone, and others, are now known to induce ICD of some tumor cells (62). Dacarbazine is the only FDA-approved chemotherapeutic agent for the treatment of melanoma, and though its use in isolation has not produced clinical benefits of major significance (63), it has been shown to promote the efficacy of a peptide-based vaccine for melanoma patients by enhancing repertoire diversity of Melan-A-specific CTL (64, 65), suggesting that the benefit of dacarbazine as part of combinatorial therapy may be derived from its induction of melanoma ICD. Likewise, mitoxantrone has been implicated in ICD in an inducible murine model of *Braf*-driven melanoma, where the antitumor effects of this chemotherapeutic were both autophagy- and T lymphocyte-dependent (66). Studies with other chemotherapeutic agents have demonstrated either direct immunogenicity of killed melanoma cells or expression/release of ICD biomarkers by melanoma cells exposed to a particular drug. In the B16-OVA model, the immunogenicity of doxorubicin-induced cell death was shown to be dependent on DC, as depletion of these cells by diphtheria toxin treatment of mice carrying the diphtheria toxin receptor transgene under control of the CD11c promoter prevented the accumulation of OVA_257_-specific CD8^+^ T cells that otherwise occurred in the lymph node draining the injection site. Although the OVA Ag in this model is more akin to a completely foreign oncoviral tumor Ag, this same study demonstrated in a humanized model of the B16-F10 murine melanoma cell line that tumor cells treated with doxorubicin and then injected into HLA-A2 transgenic hosts also conferred significant protection against a subsequent challenge with live tumor cells (67). Similarly, CD8^+^ T cell responses were also elicited against endogenous gp100 Ag in mice immunized with oxilaplatin-treated, but not live, B16-F10 cells (68). Others have also shown that lysates from oxaliplatin-treated B16-F10 melanoma cells were found to be immunogenic, conferring partial protection against subsequent challenge with live tumor cells, and this chemotherapy-driven immunogenicity was associated with markers of ICD that include cell surface calreticulin and release of ATP and HMGB1 (69). Proinflammatory cytokines/chemokines and cell surface heat shock protein 90 (HSP90) are ICD biomarkers expressed by the human A375 melanoma cell line following treatment with melphalan, an alkylating agent whose toxicity against A375 cells promoted DC maturation *in vitro*. Similar effects in the murine B78 model were also associated with *bona fide* immunogenicity *in vivo*, as vaccination with melphalan-treated tumor cells conferred complete protection against re-challenge with live cells in 40% of mice. Interestingly, this vaccination effect was independent of HSP90 expression and could be augmented by coating of melphalan-treated tumor cells with recombinant calreticulin, which was not otherwise detectable on the cell surface (70). Together, these data highlight (1) the potential for artificial delivery of DAMPs to enhance the immunogenic nature of chemotherapy-killed tumor cells but also (2) a need to better understand the role of specific ICD markers in conferring antitumor immunogenicity. Importantly, it should also be emphasized that the immunogenic potential of many of these chemotherapeutic agents has been evaluated only in prophylactic settings, and in order to achieve clinical translatability it will be necessary going forward to determine whether the immunogenicity of these regimens confers any therapeutic benefit against established tumors.

Although the expression/release of ICD biomarkers often correlates with *bona fide* immunogenicity, as was shown to be the case in many of the aforementioned studies, detection of these markers alone is not sufficient to predict immunogenicity of dying tumor cells. For instance, although mafosfamide treatment induces HMGB1 release from both EG7 lymphoma cells and B16-F10 melanoma, this cyclophosphamide derivative promotes vaccine-verified ICD only in EG7 lymphoma (71). In fact, rather than simply failing to induce immunogenicity in melanoma, cyclophosphamide has actually been suggested to promote immune suppression. Studies in the Ret transgenic melanoma model show that although low-dose cyclophosphamide induced cell surface calreticulin on skin tumor-derived Ret cells and enhanced the *in vitro* maturation of co-cultured DC, this treatment alone did not produce any survival benefit in tumor-bearing animals and even led to an accumulation of myeloid-derived suppressor cells (MDSC) in primary tumors (72). This is in contrast to the adjuvant effect that cyclophosphamide has on a DC vaccine in the MC38 colon carcinoma model, where its contribution to tumor growth inhibition correlates with an increase in cytotoxic effector infiltration of tumors and a decrease in both regulatory T cells (Tregs) and MDSC (73). Such tumor-specific differences in responsiveness to chemotherapeutic agents remain poorly understood and underscore the need to gain new insights into factors that influence tumor cell sensitivity to chemotherapy-driven ICD. Moreover, discrepancies in ICD biomarker expression and genuine ICD following tumor cell exposure to chemotherapy drugs highlight both the importance of vaccination assays as a means of verifying *bona fide* ICD as well as the significance of future studies that are necessary to evaluate the immunologic effects of DAMPs, both individually and in combination, on DC and DC-mediated immune responses so that optimal strategies for promoting robust antitumor immunity can be realized.

### Non-Chemotherapeutic Induction of ICD As a Means to Enhance Activation of Endogenous and Exogenous DC

While the aforementioned studies suggest potential utility for chemotherapy-driven ICD in promoting the immunogenicity of endogenous DC, whether this mode of ICD induction can be successful in enhancing the vaccination efficacy of exogenous DC is less clear. Combination therapy with cyclophosphamide and an autologous tumor Ag-pulsed DC vaccine has shown promise in a phase II study enrolling metastatic melanoma patients with progressive disease, but although cyclophosphamide’s effect was shown not to be the result of Treg depletion, whether its adjuvant effect was the result of ICD induction is not clear (74). Another recent phase I study has demonstrated that intratumoral injection of IFNα-differentiated unloaded autologous DC 1 day following dacarbazine treatment is associated with induction of tumor-specific CD8^+^ T cell responses and stabilization of disease in a small cohort of stage IV melanoma patients (75). Despite these hints of success, though, there is concern by many investigators that multiple cycles of chemotherapy are incongruent with the potential immunologic benefits of DC vaccination due to the lymphoablative effects of such drugs. Moreover, chemotherapeutic induction of ICD in tumor cells prior to Ag loading of DC during the production of vaccines has the potential for cytotoxicity against DC and could lead to the unintended administration of residual chemotherapeutics to vaccinated patients (49).

A number of non-chemotherapeutic interventions that overcome these limitations have been investigated for their ability to induce ICD of melanoma. Various antimicrobial/oncolytic peptides have been shown to trigger DAMP release by killed melanoma cells and promote antitumor immune responses (76, 77). Oncolytic virus therapies that take advantage of the tumoricidal potential of measles virus, vaccinia virus, and reovirus have all been shown to induce melanoma ICD as well. Specifically, studies with these oncolytic viruses have shown that infected human melanoma cells or tumor-conditioned media from these cells promote the maturation of mDC *in vitro* (78–80), and Zhang et al. have shown in a murine model that an oncolytic adenovirus co-expressing IL-12 and GM-CSF enhances the immunogenicity and antitumor efficacy of a bone marrow-derived DC (BMDC) vaccine (81). Although ICD in the context of targeted therapy for melanoma has not been thoroughly investigated, one study has shown that vemurafenib can promote cell surface exposure of calreticulin and HSP90 on various human melanoma cell lines. This same study also demonstrated that MEK inhibition could trigger exposure of these ICD biomarkers on the surface of vemurafenib-resistant melanoma cells, and tumor cells pretreated with these targeted drugs were able to promote the maturation of co-cultured DC (82). Based on these findings, it will be of interest going forward to assess how cancer immunization strategies might be coupled with targeted therapy to invoke anti-melanoma immune responses following drug-induced tumor cell death, an outcome that could result in immune-mediated eradication of tumor cells that might otherwise eventually acquire drug resistance. Finally, physical modalities that disrupt tumors, such as radiation, photodynamic therapy (PDT), high hydrostatic pressure, and hyperthermia, have been investigated for their ability to induce ICD-mediated activation of DC. Many of these approaches have been incorporated into DC vaccination setups and are currently being assessed in clinical trials for prostate cancer, ovarian cancer, and head and neck squamous cell carcinoma (83). However, melanoma resistance to many of these modalities has made their incorporation into combinatorial DC-based therapies a particular challenge. Melanoma’s relative resistance to radiotherapy is well-documented (84), and many melanomas are also resistant to PDT as a result of optical interference by melanin in pigmented tumors, the antioxidant effect of melanin, sequestration of photosensitizers in melanosomes, and other mechanisms (85). Nevertheless, interest remains in (1) exploring strategies that might sensitize melanoma cells to these physical modalities and (2) identifying particular patient populations whose melanomas might be more susceptible to these types of physical disruptions. For instance, there is evidence that depigmented melanomas are more susceptible to PDT, meaning that at least a subset of melanoma patients might benefit from PDT/DC-based combination therapies, and interventions that result in even temporary depigmentation of melanomas have the potential to increase the percentage of patients who may benefit from such combinatorial regimens (86). Along with the diverse repertoire of ICD inducers known to be effective against melanoma (Table 1), ongoing efforts to refine the use of physical modalities for tumor destruction will increase the array of weapons that exhibit not only direct antitumor activity but also the ability to boost immune reactivity against living melanoma cells, thus doubling the impact of therapy. Importantly, further optimization of therapeutic strategies with these and newly discovered ICD inducers in the future offers promise for enhancing not only naturally generated antitumor immune responses in melanoma patients but also DNA/RNA- and peptide/protein-based melanoma vaccines whose immunogenicity relies on endogenous DC to process and present Ag to tumor-specific T lymphocytes. Moreover, as is already being done with some of the aforementioned inducers of melanoma ICD, investigating how ICD inducers might maximize the immunogenicity of exogenous DC, either through *ex vivo* activation of these cells prior to immunization or through *in vivo* maintenance of their immunogenicity following infusion, will likely improve the quality and outcome of antitumor immune responses achieved by DC vaccines in future melanoma patients.

**Table 1:** Inducers of immunogenic cell death (ICD) in melanoma.

	Model system	ICD biomarker(s)	*Bona fide* ICD^a^	Reference
**Chemotherapies**				
Doxorubicin	B16-F10	Not determined	Yes	(67)
Oxilaplatin	B16-F10	Calreticulin, ATP, high-mobility group box 1 (HMGB1)	Yes	(68, 69)
Melphalan	A375	IL-8, CCL2, heat shock protein 90 (HSP90)	Not tested	(70)
	B78	HSP90	Yes	
Lidamycin	B16-F1	Calreticulin	Yes	(207)
R2016 heterocyclic quinone	B16-F10	Calreticulin, HMGB1, HSP60, HSP70, HSP90	Not tested	(208)
Ginsenoside Rg3	B16-F10	Calreticulin, HSP60, HSP70, HSP90	Not tested	(209)

**Antimicrobial/oncolytic peptides**				
LTX-315	B16-F1	HMGB1	Not tested	(76)
LTX-401	B16-F1	HMGB1, ATP, cytochrome c	Not tested	(77)

**Oncolytic viruses**				
Measles virus	Primary melanoma cells	IL-6, IL-8	Not tested	(78)
	Mel888, Mel624, MeWO, SkMel28	IL-6, IL-8, type I IFN, HMGB1		
Vaccinia virus	SK29-MEL	HMGB1, calreticulin (strain-dependent)	Not tested	(79)
Reovirus (type 3 Dearing strain)	Mel888, Mel624, MeWO, SkMel28	Proinflammatory cytokines (cell line-dependent)	Not tested	(80)

**Targeted therapies**				
Vemurafenib	A375, 451-LU, M1617	Calreticulin, HSP90	Not tested	(82)
U0126 (MEK inhibitor)	A375, 451-LU, M1617	Calreticulin, HSP90	Not tested	(82)
Bortezomib	A375, 451-LU, M1617	Calreticulin, HSP90	Not tested	(82)

**Physical modalities**				
Hyperthermia ± ionizing radiation	B16-F10	HMGB1, HSP70	Not tested	(210)

*^a^*Bona fide* ICD can be verified only in murine tumor models, as it is determined by vaccination assays in which tumor cells killed by a particular agent *in vitro* are tested for their ability to invoke protective immunity against subsequent re-challenge with live tumor cells. ICD biomarkers are indicated only if detected in a context appropriate for ICD (i.e., cell surface calreticulin and heat shock proteins, secreted ATP and HMGB1, etc.)*.

## Interfering with Immunosuppressive Networks That Impair the Function of Tumor-Associated DC

### Melanoma-Associated Suppression of DC Differentiation

A significant body of evidence now exists demonstrating that tumor cells as well as other immunosuppressive cell populations that accumulate within the tumor microenvironment produce a variety of factors that alter the function of DC (87). In the context of melanoma, such factors have been shown to interfere with the development of DC from hematopoietic precursors, to suppress the maturation and activation of already-differentiated DC, and to induce the differentiation of regulatory DC with tumor-promoting functions. In terms of DC development, hyperactivation of the STAT3 and MAPK signaling pathways has been observed in progenitors that fail to differentiate into DC in the presence of melanoma-derived factors (88), and several groups have identified specific inhibitors contributing to melanoma-associated suppression of DC differentiation. Cyclooxygenase (COX)-derived prostanoids in primary melanoma-conditioned media have been shown to inhibit the differentiation of DC from both monocytes and CD34^+^ progenitors (89). Likewise, gangliosides from human melanoma tumors impair the differentiation of DC from monocytic precursors and promote the apoptosis of monocyte-derived DC (90). A similar apoptotic effect of melanoma-derived gangliosides has also been observed on epidermal Langerhans cells (91). In addition to inhibiting the generation and viable maintenance of distinct DC subtypes, melanoma-derived factors can also skew the differentiation of DC precursors toward other myeloid populations with immunosuppressive function. For instance, TGFβ1 in B16-F10 tumor-conditioned media is capable of preventing DC differentiation from bone marrow precursors and instead drives MDSC differentiation through upregulation of the Id1 transcriptional regulator (92). COX-2-driven prostaglandin E2 (PGE_2_) in supernatants of cultured human melanoma cell lines can also promote MDSC differentiation from monocytes (93). Alternatively, macrophages capable of suppressing CD4^+^ and CD8^+^ T cell proliferation have been differentiated from monocytes cultured in conditioned media from both metastatic and non-metastatic human melanoma cell lines (94), and IL-10, which can be secreted at high levels by melanomas (95), has been shown to promote the trans-differentiation of monocyte-derived DC into tolerogenic CD14^+^ BDCA3^+^ macrophage-like cells similar to those known to be enriched in melanoma metastases (96). As immunosuppressive M2-like tumor-associated macrophages often accumulate in melanoma-bearing hosts (97–99), it is interesting to speculate that these cells may arise from an influence of tumor-derived factors on the differentiation of DC *in vivo* as well. Taken together, these influences of melanoma-derived factors on DC differentiation cannot only interfere with Ag presentation and the induction of anti-melanoma immune responses, but they can also lead to active suppression of such immune responses against melanoma.

### Melanoma-Associated Suppression of DC Maturation and Activation

In addition to its influence on the differentiation of DC, melanoma has also been shown to modulate the maturation/activation of already-differentiated DC as well. Importantly, although the presence of mature DC within tumors and tumor-draining lymph nodes is a positive prognostic factor in melanoma patients, immature DC are often enriched in both melanoma lesions and tumor-draining lymph nodes of hosts with progressive disease (100–104), thus highlighting the significance of DC maturation status as a key determinant of the immunologic control of melanoma progression. Immune dysfunction stemming from melanoma-associated effects on DC maturation and activation may result from defects in Ag processing and presentation (103, 105, 106) as well as diminished expression of costimulatory molecules and immunostimulatory cytokines, such as IL-12 (107–109). While an immature phenotype of tumor-associated DC may reflect a simple failure of tumor cells to support DC maturation and activation, active regulation of these processes by melanoma-derived factors has also been documented by several investigators. We have shown that tumor-conditioned media from murine melanoma cell lines suppresses costimulatory molecule expression and alters cytokine/chemokine expression profiles of multiple LPS-treated DC lines (110, 111), and our recent work has extended these observations to tissue-resident DC as well (99). This latter study has shown that the extent to which DC function is altered by melanoma-derived factors is tumor-dependent, such that LPS-induced costimulatory molecule expression on splenic DC-stimulated *ex vivo* as well as on lung tissue-resident DC in mice harboring melanoma lung metastases is suppressed by the rapidly progressing B16-F1 melanoma but not the poorly tumorigenic D5.1G4 melanoma. Moreover, we found that alterations to cytokine/chemokine expression profiles by DC in these systems also correlated with melanoma tumorigenicity and were partially driven by tumor-derived TGFβ1 and VEGF-A. Others have reported that immature tumor-infiltrating DC isolated from B16-F0 tumors are refractory to *ex vivo* stimulation with a cocktail of maturation stimuli but can be induced to undergo maturation following stimulation in the presence of an anti-IL-10R neutralizing antibody (112). Recently, Zelenay et al. employed CRISPR-Cas9 gene editing technology to demonstrate that COX-derived PGE_2_ in a BRAF^V600E^ melanoma cell line also suppresses costimulatory molecule expression on CD103^+^ and CD103−, CD11b^+^ tumor-infiltrating DC as well as IL-12p40 expression by the CD103^+^ DC subset (113). In addition to these studies that have elucidated roles for extrinsic tumor-derived factors in the regulation of DC maturation and activation, studies from others have provided insights into dysregulated signaling pathways within tumor-associated DC that impact these processes as well. Upregulation of β-catenin, which has been reported in DC that mature but that fail to fully activate and secrete proinflammatory cytokines (114), has been observed both in DC from lymph nodes draining B16-F10 tumors and in splenic DC cultured with B16-F10-conditioned media, and its induction in tumor-associated DC suppresses their ability to cross-prime CD8^+^ T cells (115). Similarly, impaired DC activation as measured by IL-12 secretion has been associated with hyperactivation of both the STAT3 and MAPK signaling pathways in monocyte-derived DC exposed to conditioned media or tumor lysates from human melanomas (108, 116). Most recently, upregulation of the microRNA miR148-a in tumor-associated DC was shown to impair TLR-mediated maturation by suppressing expression of the DNA methyltransferase DNMT1, which in turn led to hypomethylation of the *Socs1* gene and upregulation of the SOCS1 TLR signaling suppressor (117).

### Melanoma-Associated Induction of Regulatory DC Function

Beyond limitations on the Ag processing/presentation and maturation/activation capacity of DC that can preclude induction of antitumor immunity and lead to tumor immune tolerance, respectively, melanoma-derived factors have also been shown to trigger development of regulatory DC with various tumor-promoting functions. Such DCs have been shown to contribute to tumor angiogenesis (118), the development and recruitment of immunosuppressive Tregs (119–121), and the direct suppression of CD4^+^ and CD8^+^ T cells (122, 123). Importantly, several studies have now provided mechanistic insights into both the induction of regulatory DC and the tumor-supporting activities mediated by these cells. One study has reported upregulation of the PD-L1 co-inhibitor that dampens CD8^+^ T cell effector function on tumor-infiltrating DC in the B16-F10 model (124). Another study has shown that melanoma-derived IL-10 and other unidentified factors contribute to an IL-12^low^, IL-10^high^ phenotype in monocyte-derived DC capable of inducing CD4^+^ CD25^+^ FOXP3^+^ Treg development (125), and tumor-derived IL-6, VEGF, and TGFβ1 have all been implicated in the induction of IL-12^low^, IL-10^high^ DC in the spontaneous Ret murine melanoma model (126). Differentiation of IL-10-producing regulatory DC has also been shown to be driven by autocrine IL-6/IL-10 signaling through STAT3 in DC, which is initiated by melanoma-derived factors that activate the TLR2 signaling pathway in these cells (127). Additionally, Treg expansion in melanoma can also be driven by TGFβ1-producing regulatory DC (128). Still others have found that regulatory DCs produce enzymes that diminish the availability of metabolites crucial for T cell activation, thereby inducing metabolic suppression of anti-melanoma immunity. In particular, mDC that were imprinted by ER stress in melanoma cells suppressed CD8^+^ T cell proliferation *via* secretion of the arginine-depleting enzyme arginase I (123), and melanoma-educated regulatory DCs have also been found to suppress CD4^+^ T cell proliferation in an arginase-dependent manner (122). Likewise, tryptophan catabolism by indoleamine 2,3-dioxygenase (IDO)-producing regulatory pDC recovered from melanoma-draining lymph nodes is associated both with suppression of CD8^+^ T cells (129) and with activation of CD4^+^ Tregs (130). In addition to this IDO-mediated regulation of anti-melanoma immunity, regulatory pDC have also been shown to drive T_H_2 and Treg differentiation of CD4^+^ T cells through cell–cell interactions *via* OX40L and ICOSL, respectively (131).

### Strategies to Overcome Melanoma-Associated Dysregulation of DC Function

**Figure 2:**
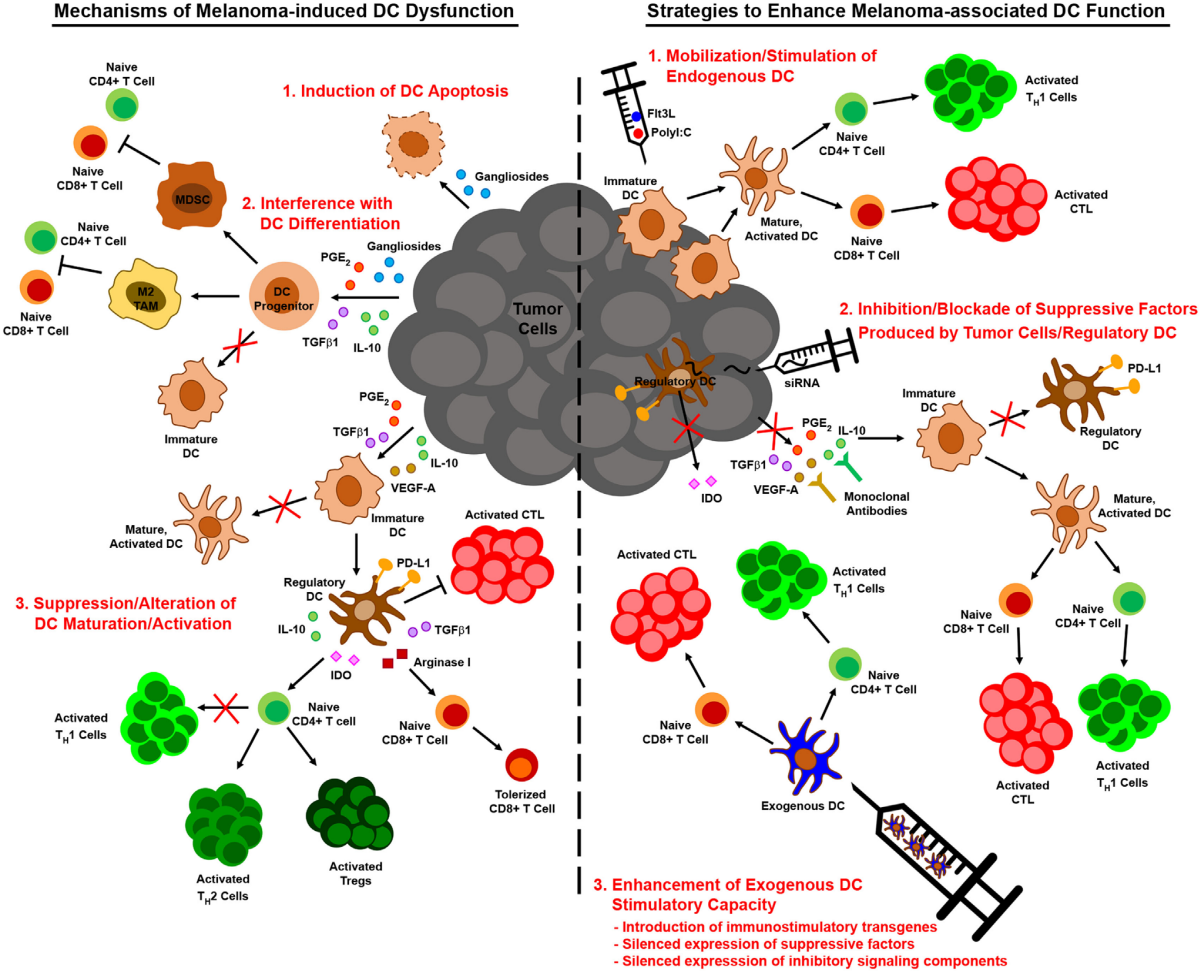
Melanoma-associated dendritic cell (DC) dysfunction and therapeutic interventions to enhance DC-mediated anti-melanoma immune responses. Melanoma interferes with the function of DC by numerous mechanisms, including induction of DC apoptosis, blocking/altering DC development from hematopoietic precursors, suppressing DC maturation/activation, and driving the differentiation of tumor-promoting regulatory DC (left). Insights into these mechanisms of melanoma-altered DC dysfunction have informed strategies to augment DC-mediated anti-melanoma immune responses. These strategies include approaches that mobilize and stimulate endogenous DC, interventions that impede the production or action of immunosuppressive factors released by melanoma and associated cells in the tumor microenvironment, and regimens that employ exogenous DC that have been manipulated to resist suppressive elements and stimulate robust antitumor immunity (right).

While the previously described studies highlight diverse mechanisms by which melanoma may subvert DC-mediated antitumor immunity, insights into melanoma-altered DC function have suggested novel strategies for improving DC-based immunotherapies for this cancer (Figure 2). To overcome the paucity and poorly immunogenic nature of DC within melanoma lesions, strategies to increase tumor infiltration by DC and promote their activation *in situ* have shown promise in murine melanoma models. Salmon et al. recently demonstrated that systemic administration of Flt3L expanded and mobilized CD103^+^ DC progenitors from the bone marrow and led to the accumulation of immature CD103^+^ DC within tumor masses, and subsequent injection of polyI:C intratumorally induced local maturation of these cells and enhanced their ability to recruit and activate melanoma-specific effector CD8^+^ T cells, leading to tumor regression (47). Similar findings were recently reported by Sánchez-Paulete et al., who demonstrated that Flt3L-mobilized Batf3-dependent DC activated by poly-ICLC synergized with anti-CD137 and anti-PD-1 monoclonal antibody therapy to promote Ag-specific CD8^+^ T cell cross-priming and tumor control (132). Likewise, Tzeng et al. found that administration of IFNα (as well as other DC maturation stimuli) after treatment of melanoma-bearing mice with a combination therapy that mediates tumor Ag release enhanced the cross-presentation and cross-priming activities of CD8α^+^ DC in tumor-draining lymph nodes (133). Importantly, although this maneuver led to complete regression of established tumors in a large percentage of mice, minimal benefit was observed when IFNα was administered either before or concomitantly with combination therapy, as the loss of phagocytic capacity that accompanied CD8α^+^ DC maturation at these early times limited the ability of these cells to acquire tumor Ag later released as a result of therapy. These data thus highlight the importance of treatment schedule and the temporal programming of DC maturation/activation in combinatorial approaches that rely on endogenous DC to trigger therapy-associated antitumor immune responses. Early clinical studies demonstrating that it is also possible to directly manipulate the frequency and maturation status of endogenous DC in melanoma patients have also reinforced the need for optimizing strategies to maximize the immunogenicity of these cells. For instance, local administration of a mix of CpG-B and GM-CSF at the site of primary melanoma excision resulted in the maturation of both pDC and conventional DC as well as an increase in the frequency of cross-presenting BDCA3^+^ CD141^+^ DC in sentinel lymph nodes, and this approach enhanced the frequency of melanoma Ag-specific CD8^+^ T cells in these nodes and reduced the frequency of lymph node metastasis (134, 135). At the same time, though, this approach also enhanced the suppressive activity of CD4^+^ Tregs in sentinel lymph nodes, suggesting that further optimization of this regimen may enable more robust antitumor immunity and even better clinical results. The identification of optimal DC stimulation cocktails and the implementation of combinatorial regimens that offset the deleterious activities of *in situ*-stimulated DC are therefore critical areas of investigation that may drive the development of more efficacious anti-melanoma immune therapies in the future. Moreover, advances in targeted delivery of therapeutics to endogenous DC, such as those that have already been achieved with IDO siRNA-encapsulated mannosed liposomes (136) and polypeptide micelle-based nanoparticles incorporating an miRNA148-a inhibitor (117), will enable selective reprogramming of melanoma-associated DC into potent stimulators of antitumor immune responses and likely improve the outcome of immunotherapy for melanoma patients going forward.

In contrast to strategies aimed at improving the immunogenicity of endogenous melanoma-associated DC, approaches to enhance the immunostimulatory capacity of exogenous DC have also improved the efficacy of many melanoma vaccines. For example, strategies that provide immune stimulating support for exogenous DC, such as the introduction of IL-6 or IL-21 transgenes into BMDC (137, 138) or the co-administration of oncolytic adenovirus engineered to express immune stimulators such as IL-12 and GM-CSF (64, 119), have been shown to significantly improve vaccine efficacy, resulting in complete regression of established melanomas in some cases. Combinatorial approaches that aim to neutralize the effects of tumor-derived factors on exogenously administered DC, such as local siRNA-mediated silencing of TGFβ1 at the tumor site (139), have also been effective. Alternatively, manipulation of exogenous DC prior to immunization by gene-silencing approaches can promote the immunostimulatory capacity of these cells in two ways. First, silencing the expression of genes involved in signaling pathways that limit the immunostimulatory function of melanoma-associated DC can prevent their immunosuppression by tumor-derived factors. In this regard, vaccines employing SOCS1-silenced DC improve the control of established B16 melanoma (140, 141), a finding that offers exciting proof-of-principle for this approach and that suggests the silencing of other immunosuppressive signaling molecules often dysregulated in melanoma-altered DC, such as STAT3 and β-catenin, may also improve the antitumor efficacy of DC vaccines for melanoma. Second, silencing the expression of suppressive factors known to be released by melanoma-induced regulatory DC can prevent conversion of exogenous DC from immune activating cells to immunosuppressive ones. Indeed, vaccination of mice with IDO-silenced DC confers partial protection against B16 melanoma (142), and a recent case report has revealed immunologic and clinical benefits of an IDO-silenced DC vaccine in a melanoma patient (143). Similarly, *in vitro* studies have shown that IL-10-silenced human mDC are better able to elicit CTL activation against an antigenic epitope of MART-1 (144), suggesting that immunization with such DCs might improve antitumor immunity in melanoma patients as well. Altogether, these and related strategies for improving the function of DCs in the context of melanoma offer exciting promise for DC-based immunotherapies designed to overcome melanoma-imposed limitations on these cells and the antitumor immune responses they mediate.

## Overcoming Metabolic Constraints on DC Function within the Tumor Microenvironment

### Metabolic Reprogramming of DC and Tumor Cells

The emerging role of immunometabolism in the regulation of DC function in recent years has revealed new mechanisms by which tumors may subvert DC-mediated antitumor immunity. Indeed, beyond the aforementioned mechanisms of tumor-associated immunosuppression of DC, metabolic suppression of DC in the tumor microenvironment is now recognized as a significant barrier to DC function, which is controlled by key metabolic pathways regulating the bioenergetic and biosynthetic needs of these cells. While immature DC in the steady state rely on fatty acid oxidation and oxidative phosphorylation (OXPHOS) as their primary modes of metabolism, TLR-stimulated DC undergo a metabolic switch to aerobic glycolysis within minutes of the maturation and activation process (145). This early switch to glycolytic metabolism provides a source of carbon for the pentose phosphate pathway and tricarboxylic acid (TCA) cycle, both of which produce intermediates for fatty acid synthesis needed to support the expansion of membrane mass for the ER and Golgi apparatus, thus allowing DC to meet the demands of protein synthesis, transport, and secretion that are associated with maturation/activation (146). Long-term commitment to glycolytic metabolism in activated DC then fuels ATP production and survival in the face of decreasing mitochondrial metabolism, which results from OXPHOS inhibition by nitric oxide in inflammatory DC (147) and from autocrine type I IFN induction of the HIF1α transcription factor that blocks mitochondrial respiration in conventional DC (148). Interestingly, metabolic suppression of tumor-associated DC is often a consequence of metabolic reprogramming in tumor cells themselves, which are driven by the activation/deactivation of oncogenes/tumor suppressor genes and harsh environmental conditions (such as hypoxia) to switch from OXPHOS to glycolysis as the primary mode of metabolism, in this case to support the energy and biosynthetic demands of rapidly proliferating cells. Indeed, even under normoxic conditions, tumor cells are reprogrammed for a primarily glycolytic-based mode of energy production (aerobic glycolysis, otherwise known as the “Warburg effect” in tumor cells), thus allowing intermediates of the glycolytic pathway to function as important metabolites for macromolecule biosynthesis by mitochondria no longer relied as heavily upon for OXPHOS (149–151). Therefore, as metabolically reprogrammed tumors grow, their increasing demand for glucose consumption contributes to an environment that is metabolically hostile to infiltrating DC and other immune cell populations, with competition for limiting nutrients and accumulation of toxic metabolic byproducts released by tumor cells into the extracellular space both impairing immune system function.

### Metabolic Suppression of DC in the Context of Melanoma

In melanoma, metabolic rewiring for glycolysis may be driven by multiple signaling pathways, including BRAF-driven MAPK hyperactivation that negatively regulates OXPHOS (152) and PI3K/AKT/mTOR/HIF1α signaling that positively regulates glycolysis (153). These signaling pathways induce expression of glucose transporters as well as enzymes that favor glycolytic metabolism, such as lactate dehydrogenase A (LDHA) that converts the glycolysis end-product pyruvate into lactic acid, thus diverting pyruvate from utilization in the TCA cycle as fuel for OXPHOS (154). Importantly, depletion of glucose in the tumor microenvironment by melanomas exhibiting high glycolytic activity may impair glycolysis, and in turn ATP production, in tumor-infiltrating DC. Such effects may alter the AMP:ATP ratio in DC and lead to AMP-mediated activation of the nutrient/energy sensor AMPK (155), which is known to promote OXPHOS and suppress mTOR and HIF1α signaling (156–158), thus further contributing to the negative regulation of glycolysis in these cells. Beyond the effects of glucose deprivation in the tumor microenvironment on DC function, buildup of lactic acid in the extracellular space of glycolytically active melanomas can also suppress DC. In this regard, melanoma-derived lactic acid inhibits the differentiation of monocyte-derived DC and suppresses IL-12 production by previously differentiated monocyte-derived DC stimulated with LPS *in vitro* (159). Although the mechanism by which lactic acid influences tumor-associated DC function has yet to be elucidated, there is speculation that altered membrane transport in the lactate-rich tumor microenvironment might contribute to its suppressive effect (160, 161). Because lactate is transported passively by facilitated diffusion through monocarboxylate transporters, high levels of extracellular lactate within the tumor microenvironment might promote import of melanoma-derived lactic acid into DC while at the same time precluding export of lactic acid produced within DC also undergoing aerobic glycolysis, leading to a buildup of lactate within DC that impairs the glycolytic flux necessary to maintain an activated phenotype. Alternatively, lactate was recently shown to inhibit macrophage activation by binding to the GPR81 lactate receptor and suppressing TLR signaling (162), and it is possible that this pathway might also contribute to lactate-associated suppression of DC stimulated by tumor-derived DAMPs. Finally, evidence is emerging that suppression of glycolysis in DC is not merely a consequence of the metabolic limitations imposed by glycolytically active tumor cells, as tumor-derived immunosuppressive cytokines have also been shown to alter DC metabolism. For instance, IL-10 was found to suppress the metabolic switch to aerobic glycolysis in LPS-stimulated DC by antagonizing TLR ligand-mediated hypophosphorylation of AMPK (145). Similarly, IL-10 is known to promote *Socs3* gene expression (163), and melanoma-associated DC have been found to exhibit SOCS3-mediated inhibition of the M2 pyruvate kinase (PKM2) that catalyzes conversion of phosphoenolpyruvate into pyruvate in the final step of glycolysis (164).

In addition to the key role played by glycolytic metabolism in the activation of DC, the metabolism of fatty acids has also been shown to be an important regulator of DC function. Although lipid synthesis is important for ER and Golgi biogenesis during DC activation, the accumulation of lipids in DC in the context of cancer is often associated with immune dysfunction. In particular, Herber et al. demonstrated that several species of triglycerides accumulate in DC cultured with various tumor explant supernatants, including that of B16-F10 melanoma, and that high lipid content in tumor-associated DC impaired tumor Ag processing and cross-presentation (165). Interestingly, DC cultured with tumor-derived supernatant also exhibited increased expression of the scavenger receptor MSR1, suggesting that the accumulation of lipids in these DCs might arise from tumor-derived factors that promote DC uptake of fatty acids in the form of lipoproteins, as triglycerides are typically not taken up by DC but can be synthesized from lipoprotein precursors within cells. Subsequent studies revealed that lipid accumulation in tumor-associated DC defective in cross-presentation resulted from an increase in polyunsaturated fatty acids, particularly linoleic acid and to a lesser extent arachidonic acid, and that DC isolated from tumor-bearing mice or exposed to tumor explant supernatants *in vitro* exhibited significantly higher levels of oxidized free fatty acids and oxidatively truncated triglycerides (166). Of note, these DC did not exhibit oxidation of phospholipids that would be a major component of ER and Golgi membranes. These data may therefore explain the apparent discrepancy between the need for DC to undergo *de novo* lipogenesis to support ER and Golgi biogenesis during activation and the dysfunction that results from lipid accumulation in the context of tumors, suggesting that it is the nature and oxidation status of the fatty acids accumulating in tumor-associated DC that is detrimental to their function. Indeed, oxidized fatty acids have been shown to inhibit DC maturation through binding and activation of the peroxisome proliferator-activated receptor PPARγ, which promotes fatty acid synthesis and storage (167). Additionally, others have reported that lipid peroxidation by reactive oxygen species within tumor-associated DC yields byproducts that upregulate the ER stress sensor XBP1, which activates genes involved in the biosynthesis and accumulation of triglycerides known to be linked with DC dysfunction (168). Altogether, these studies reveal the complex regulation of lipid metabolism that controls DC function, and they highlight how factors in the tumor microenvironment can alter this process to ultimately promote tumor immune escape.

While alterations to glycolysis and lipid metabolism impair tumor-associated DC function by influencing how major macromolecules necessary for cell survival and activation are utilized, other metabolites that frequently accumulate in the tumor microenvironment are also known to compromise the function of DC and DC-mediated immune responses. Adenosine is a particularly well-characterized metabolite that accumulates in the extracellular space of many tumors, including melanoma (169). Although ATP released from tumor cells may serve as a DAMP to promote DC activation (see Induction of Immunogenic Cell Death (ICD) as a Means of Promoting DC-Mediated Antitumor Immunity), melanoma cells often express on their surface the CD39 and CD73 ectonucleotidases that hydrolyze ATP into adenosine (170–172), thereby leading to its buildup in the tumor microenvironment. In addition to its role in the suppression of T cell signaling (173) and immunosuppressive activity of Tregs (174), adenosine has also been shown to impair DC function. *In vitro* studies with LPS-stimulated human monocyte-derived DCs have shown that adenosine promotes IL-10 secretion while suppressing IL-12 and TNFα secretion as well as the capacity of DC to promote T_H_1 differentiation (175). Others have shown that DC differentiated from monocytic precursors in the presence of adenosine acquire several tumor-promoting functions that are dependent on signaling through the A_2B_ adenosine receptor. These pro-tumor functions include increased expression of angiogenic factors, immunosuppressive cytokines, and proteins that disrupt immunometabolism such VEGF, TGFβ, IDO, and arginase 2, among others (176). In the context of melanoma, *in vivo* studies in B16-F10 tumor-bearing mice have shown that adenosine signaling through the A_2A_ adenosine receptor on DC is associated with a slight decrease in MHC II and IL-12 expression and a significant increase in the expression of IL-10 (177). Interestingly, recent studies have shown that adenosine receptor signaling in DC also promotes accumulation of intracellular cAMP (178), suggesting that adenosine may ultimately suppress DC activation by influencing AMPK activity and decreasing glycolytic metabolism in these cells. Finally, whereas melanoma cells are one of the major sources of adenosine in the tumor microenvironment, immunoregulatory metabolites that compromise DC function may also be produced by other cell types known to infiltrate tumors. For instance, arginase I-producing cells such as MDSC produce ornithine as a byproduct of arginine metabolism, and ornithine decarboxylation yields polyamines that enhance IDO-1 expression in DC, thus conditioning these cells for immunosuppressive activity (179). Even melanoma-associated DC themselves can contribute immunosuppressive metabolites to the extracellular milieu of progressive tumors. Specifically, melanoma-induced activation of β-catenin signaling in DC from tumor-draining lymph nodes promotes expression of enzymes involved in vitamin A metabolism, leading to DC secretion of the vitamin A metabolite retinoic acid that in turn promotes differentiation of immunosuppressive Tregs (120). Collectively, these studies highlight the metabolically hostile nature of the tumor microenvironment that must be overcome in order for DC to elicit and maintain effective antitumor immune responses.

### Metabolic Interventions to Promote DC Function in the Context of Melanoma

**Figure 3:**
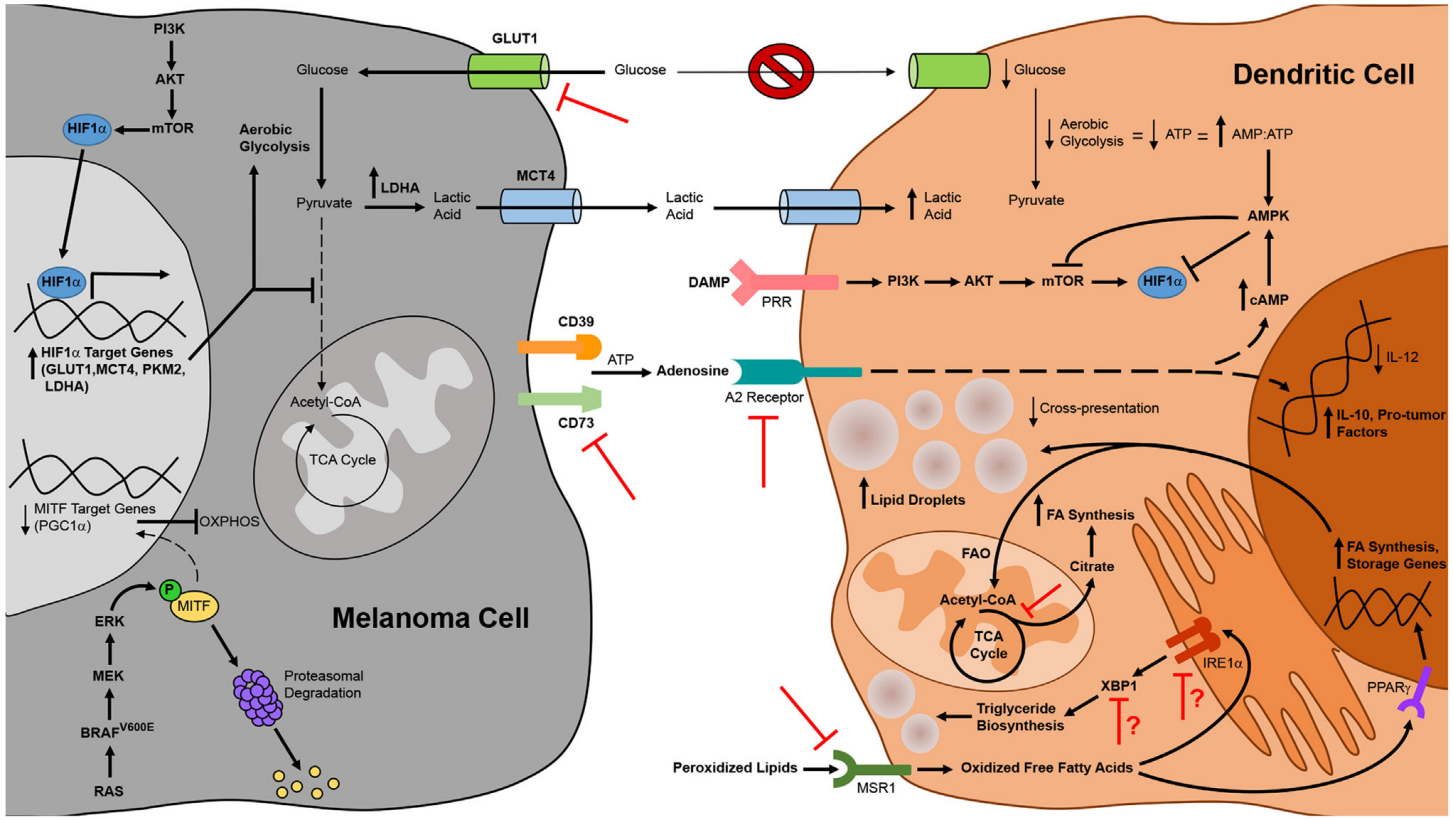
Alterations to tumor cell and dendritic cell (DC) metabolism in the context of melanoma and therapeutic strategies to overcome metabolic suppression of melanoma-associated DC. DC function in the context of melanoma is compromised by constraints on metabolic pathways essential to DC maturation and activation. Metabolic suppression of DC in the tumor microenvironment arises from nutrient depletion and the buildup of toxic waste that results from metabolic rewiring of melanoma cells for aerobic glycolysis. Uptake of peroxidized lipids within the tumor microenvironment also promotes DC dysfunction, leading to an accumulation of oxidized lipids in DC that impairs cross-presentation of tumor antigen. Additionally, tumor cell release of immunosuppressive metabolites such as adenosine that signal through DC inhibit the antitumor function of these cells. Mechanistic insights into these phenomena have identified novel targets for therapies designed to interfere with metabolic pathways in melanoma cells or to prevent tumor-altered metabolism of melanoma-associated DC. Pharmacologic interventions or tissue-specific gene-silencing approaches that target factors upregulated by melanoma cells have direct antitumor effects and are also likely to improve DC function indirectly by creating a more hospitable metabolic microenvironment. Similarly, DC-targeted delivery of therapeutics that prevent uptake of suppressive metabolites or that block metabolic pathways associated with tolerogenicity can improve the immunostimulatory function of endogenous DC, and manipulation of exogenous DC to resist the induction of metabolic suppression can improve the efficacy of DC vaccines. Bold arrows and type designate metabolic pathways, metabolites, intermediates, and processes that are elevated in melanoma cells and tumor-altered DC. Arrows and type not in bold represent those that are downregulated in these cells. Red inhibition symbols highlight proteins and metabolic pathways that have been successfully targeted in melanoma cells and DC in preclinical studies, as described in the text. Red inhibition symbols with red question marks indicate targets that have been associated with both immune activating and immune suppressing functions in different models and whose inhibition may therefore be appropriate only in certain contexts, as is discussed in more detail in the text.

Just as insights into melanoma-associated immune suppression of DC have informed therapeutic strategies to enhance the immunogenicity of these cells, so too have insights into the metabolic suppression of melanoma-associated DC (Figure 3). To overcome the immune dampening effects of retinoic acid signaling, a retinoic acid receptor α antagonist has been used to enhance the efficacy of a peptide-pulsed DC vaccine against B16 melanoma. In addition to enhancing DC production of IL-12 and lowering DC production of TGFβ and IL-10, this antagonist reduced the number of FOXP3^+^ IL-10^+^ Tregs that infiltrated tumors (180). Pharmacologic inhibition of the β-catenin/TCF pathway that promotes melanoma-associated DC production of retinoic acid has also been shown to reduce the expression of vitamin A-metabolizing genes in DC isolated from tumor-draining lymph nodes, and the antitumor activity associated with this inhibition correlated with reduced Treg and increased effector CD8^+^ T cell infiltration of subcutaneous melanomas (120). Likewise, inhibition of adenosine in the tumor microenvironment may be approached in a number of ways to prevent its deleterious effects on DC function. Pharmacological antagonists of the A_2B_ receptor block the effects of adenosine on DC differentiation *in vitro*, and DC from both A_2A_ and A_2B_ receptor knockout mice are resistant to the suppressive effects of adenosine (176, 177). Therefore, neutralization of adenosine signaling in DC *via* pharmacologic agents or gene-silencing approaches that knock down expression of adenosine receptors on either endogenous or exogenous DC might improve the antitumor immunogenicity of these cells. Alternatively, strategies that interfere with the CD73 ectonucleotidase on melanoma cells have already been shown to improve antitumor immunity in preclinical models (169, 181), and this outcome is likely due to a reduction in the immunoregulatory effects of adenosine on multiple immune cell populations, including DC.

In addition to overcoming the suppressive effects of extracellular metabolites on DC in the tumor microenvironment, maneuvers that interfere with the metabolism of macromolecules in melanoma cells and/or DC may also restore metabolic and immune function in tumor-associated DC. Pharmacologic regulation of lipid levels in DC using an inhibitor of acetyl-CoA carboxylase that blocks fatty acid synthesis improved the antitumor efficacy of a peptide vaccine against B16-F10 melanoma (165). It is also possible to regulate lipid levels in DC by targeting the MSR1 scavenger receptor that promotes lipid uptake or the IRE1α/XBP1 pathway that triggers triglyceride synthesis in tumor-associated DC. To this point, immunization of tumor-bearing mice with MSR1 gene-silenced BMDC improved vaccine-induced CD8^+^ T cell responses against multiple melanoma antigens and enhanced immunologic control of established B16 melanomas in both subcutaneous and lung metastasis models (182). Likewise, targeted delivery of nanoparticles encapsulating siRNA has been used to silence in tumor-associated DC the expression of either XBP1 or the IRE1α endoribonuclease that cleaves *Xbp1* mRNA into a form that encodes functional protein during ER stress. In a murine model of ovarian cancer, this approach reduced triglyceride levels in tumor-associated DC, augmented the activation of tumor Ag-specific T cells, and improved tumor immune control and overall survival of tumor-bearing mice (168). As triglycerides are also known to accumulate in dysfunctional melanoma-associated DC (165), silencing of IRE1α or XBP1 expression in these cells might also improve DC-mediated immune responses against this cancer in certain contexts. It is worth noting, however, that overexpression of XBP1 in BMDC actually improves DC survival, activation, and T cell stimulatory capacity, leading to enhanced immune control of established B16 melanoma following vaccination (183). Additionally, in an inducible BRAF^V600E^/PTEN-driven melanoma model, a DNA vaccine that promotes XBP1 expression in endogenous DC conferred CD8^+^ T cell-mediated immune control of small established tumors (184). While tumor microenvironment-specific differences in these ovarian cancer and melanoma models may explain differences in the impact of XBP1 on DC function, it is also possible that these discrepancies are due to differences in the particular DC under study, including the endogenous/exogenous nature of these cells and the extent of ER stress in the DC in which XBP1 is active. It is interesting to speculate that in DC which have not previously been exposed to the hostile tumor microenvironment (i.e., exogenous BMDC) or which are found in the context of early stage tumors and have not yet accumulated the types of fatty acids associated with immune dysfunction, XBP1 promotes DC immunogenicity by protecting these cells against ER stress as they increase protein synthesis during their activation. On the other hand, in endogenous DC that have incorporated significant polyunsaturated fatty acids within the microenvironment of late-stage tumors, XBP1 activation may lead to the generation of oxidized triglycerides that impair DC function. Future studies will be necessary to test this hypothesis and define the parameters under which XBP1 activation versus inactivation in DC is appropriate for optimizing the antitumor activity of these cells.

Finally, glycolytic metabolism in both melanoma cells and DC can be targeted to enhance the immunostimulatory capacity of DC. Recent studies have demonstrated that silencing of the GLUT1 glucose transporter or the CD147 gene product that regulates its expression in melanoma cell lines impairs the growth and metastasis of transplanted tumors (185, 186). In addition to having direct antitumor effects, interfering with glycolysis in melanoma cells may have pro-immune consequences as well, resulting in enhanced DC-mediated antitumor immune responses by increasing glucose availability and decreasing lactic acid concentration in the tumor microenvironment. Therefore, targeting glucose transporters and other enzymes (such as LDHA) that are involved in glycolytic metabolism in melanoma cells is a potentially attractive therapeutic option for the treatment of melanoma. While selective targeting of such therapies specifically to tumor cells might be difficult for some cancer types and could lead to compromised function of DC and other immune cell populations that also rely on glycolysis for induction and maintenance of an activated phenotype, the identification of tissue-specific genes in melanoma (such as those involved in the melanin deposition pathway) opens up the possibility of DNA-based therapies in which siRNA/shRNA expression is driven off of tissue-specific promoters active only in melanoma cells. Such a strategy would overcome issues with selective *delivery* of siRNA/shRNA to tumor cells and instead would rely on selective *activation* of a gene-silencing therapeutic specifically in melanoma cells. Alternatively, it is also possible to minimize the reliance of DC on glycolysis as the sole bioenergetic mode of metabolism during activation. Although signaling through mTOR is associated with a metabolic switch to aerobic glycolysis during DC activation as described above, this switch results less from a preference for glycolytic metabolism and more from a requirement for glycolysis as a means of generating ATP in the face of mitochondrial suppression by reactive oxygen species. Interestingly, it has been reported that inhibition of mTOR in DC does not preclude ATP synthesis in these cells and instead extends the lifespan of activated DC by reducing reactive oxygen species and preserving mitochondrial function, thus allowing flexibility in the metabolic pathways utilized by DC for bioenergetic purposes (187). Indeed, multiple groups have shown that interfering with mTOR function in BMDC enhances vaccine-induced CD8^+^ T cell responses and immunologic control of established B16 melanomas (188, 189). Together, these data highlight how metabolic interventions may shift the profile of tumor-associated DC from tolerogenic to immunogenic, and they suggest great promise for metabolism-based therapies, either alone or in combination with immunotherapies, in the treatment of melanoma.

## Modulating the Microbiome to Augment DC-Mediated Antitumor Immunity

### Gut Microbiome Influences on Natural Antitumor Immunity to Melanoma

As data have emerged demonstrating that the microbiota and dysbiosis play significant roles in both cancer progression and the efficacy of anticancer therapies (190), there has been considerable interest in understanding how the microbiome regulates the quality of antitumor immune responses. In the context of melanoma, altering the composition of the gut microbiota has been shown to impact both natural and therapy-associated antitumor immunity, and in many cases, regulation of these responses has been associated with microbial influences on DC activation. Antibiotic treatment with a mixture of ampicillin, vancomycin, and neomycin sulfate (which leads to a decreased frequency of gut bacteria belonging to the Bacteroidetes phylum and an increased frequency of gut bacteria belonging to the Firmicutes phylum) prior to B16-F10 challenge enhances tumor outgrowth and is associated with defects in natural antitumor immunity that include a decrease in the frequency of DC among tumor-infiltrating leukocytes and a reduced expression of genes associated with DC maturation and immune activation within tumor tissue (191). Addition of metronidazole to the aforementioned cocktail of antibiotics yields a different type of gut dysbiosis in treated mice (decreased frequency of both Firmicutes and Bacteroidetes phyla members and increased frequency of members of the Proteobacteria phylum), and this alteration also leads to impaired immune control of B16-F10 lung metastases (192). This latter effect results from an antibiotic-associated decrease in IL-17^+^ γδT cells in the lungs of treated mice. Although the mechanism by which microbial dysbiosis influences γδT cell function remains to be elucidated in this model, the authors speculated that a lack of DC stimulation by PAMPs in antibiotic-treated mice could contribute to the observed decrease in gene expression in the lungs of IL-6 and IL-23, cytokines known to activate IL-17 production by γδT cells.

### Gut Microbiome Influences on Therapy-Associated Immunity to Melanoma

The first study to report microbial influences on the outcome of immune therapy for cancer demonstrated that the therapeutic benefit of total body irradiation prior to adoptive T cell transfer arises in part from activation of the innate immune system following radiation-induced damage to the GI tract and subsequent translocation of gut microbiota (*Enterobacter cloacae, Escherichia coli, Lactobacillus*, and *Bifidobacterium*) to mesenteric lymph nodes (193). In addition to mobilizing the gut microbiome, total body irradiation also led to elevated serum LPS levels and an increase in the absolute number of CD86^hi^ DC in the spleen and lymph nodes, which in turn correlated with enhanced activation of adoptively transferred gp100-specific CD8^+^ T cells and improved control of established B16-F10 tumors. Interestingly, when mice were administered the broad-spectrum antibiotic ciprofloxacin beginning two days prior to irradiation, microbial translocation to lymph nodes was not observed, nor was any elevation in serum LPS levels. Likewise, the immunologic and antitumor benefits of DC and CD8^+^ T cell activation were also abrogated following ciprofloxacin depletion of gut microbiota. Additional experiments with the LPS-blocking antibiotic polymyxin B as well as TLR4^−/−^ mice revealed that the therapeutic effect of gut microbiota translocation following total body irradiation resulted from LPS stimulation of innate immune cells that support the activation of adoptively transferred CD8^+^ T cells. In related work, Iida et al. showed that treating mice with a cocktail of antibiotics (vancomycin, imipenem, and neomycin) abrogated the antitumor effects of combination immunotherapy with anti-IL-10 receptor antibody and intratumoral CpG-oligodeoxynucleotides (ODN) in B16-F10 tumor-bearing mice (194). Though the mechanistic basis for these findings was not further studied in the B16 melanoma model, the authors reported analogous findings in the MC38 colon adenocarcinoma model, where antibiotic treatment decreased both the frequency of TNF-producing tumor-infiltrating DC (and other leukocytes) as well as CD86 expression and IL-12p40 production by tumor-associated DC. Similar results were also observed following combination immunotherapy of germ-free MC38-bearing mice, suggesting that commensal microbes are necessary to prime DC and other myeloid cell populations for inflammatory cytokine production in response to this immune therapy.

More recently, the microbiome has been shown to influence DC function and antitumor immunity in the context of checkpoint blockade therapies for melanoma as well. In the B16-SIY melanoma model, the success of α-PD-L1 Ab therapy was shown to rely on the presence within the intestinal microbiota of *Bifidobacterium* species that enhance the antitumor effects of therapy (195). Specifically, the presence of natural *Bifidobacterium* species in C57Bl/6 mice from The Jackson Laboratory (JAX) or the introduction of *Bifidobacterium* species by oral gavage into C57Bl/6 mice from Taconic (TAC), which do not naturally harbor these bacteria, correlated with tumor-specific CD8^+^ T cell responsiveness to α-PD-L1 Ab therapy and tumor control. Of note, the presence of intestinal *Bifidobacterium* species in these mice was also associated with an increase in the frequency of intratumoral DC expressing high levels of MHC class II, and genome-wide transcriptional profiling of these cells revealed elevated expression of several genes known to play roles in DC maturation, Ag processing and presentation, costimulation, and chemokine-mediated recruitment of immune effectors. Moreover, DC isolated from lymphoid tissues of JAX mice and *Bifidobacterium*-fed TAC mice induced higher levels of IFNγ production by CD8^+^ T cells than did DC from untreated TAC mice that had not been exposed to *Bifidobacterium* species. In other work investigating microbial influences on checkpoint blockade therapy, pretreatment of mice with a cocktail of broad-spectrum antibiotics blocked the efficacy of α-CTLA-4 Ab therapy for established Ret murine melanomas (196). Interestingly, in mice not treated with antibiotics, CTLA-4 blockade promoted T cell-mediated destruction of intestinal epithelial cells and was associated in general with a decrease in Bacteroidales and Burkholderiales member species and an increase in Clostridiales member species in the feces, suggesting that induction of immunity to members of the Bacteroidales and Burkholderiales orders may be linked to the induction of antitumor T cell responses. In this regard, antibiotic-treated or germ-free mice that otherwise failed to exhibit any antitumor effects following α-CTLA Ab therapy were able to control tumors when fed with *Bacteroides thetaiotaomicron, Bacteroides fragilis, Burkholderia cepacia*, or a combination of *B. fragilis* and *B. cepacia* shortly after therapy, and this response was associated with enhanced maturation of intratumoral DC and T_H_1 immune responses in tumor-draining lymph nodes. Moreover, fecal transplantation studies in which feces from ipilimumab-treated metastatic melanoma patients clustered by stool microbial composition were transferred to germ-free mice two weeks prior to tumor challenge and α-CTLA-4 Ab therapy supported a role for *Bacteroides* species in promoting responsiveness to therapy. In these studies, feces from only one cluster of melanoma patients promoted colonization of immunogenic *B. thetaiotaomicron* and *B. fragilis* in mice, and these animals were the only fecal transplant recipients to mount effective antitumor responses following α-CTLA-4 Ab treatment. While these data suggest that the presence of commensal *Bacteroides* species in the gut may be a useful prognostic indicator for identifying patients most likely to benefit from checkpoint blockade therapy, it should be noted that confounding data on the influence of *Bacteroides* species on therapeutic efficacy in metastatic melanoma patients have emerged from recent clinical studies. Indeed, in a prospective study of metastatic melanoma patients receiving ipilimumab therapy, a high proportion of baseline gut *Bacteroides* actually correlated with poor clinical benefit, whereas long-term benefit (progression-free and overall survival) was associated with enrichment of *Faecalibacterium* species and other Firmicutes phylum members (unclassified *Ruminococcaceae, Clostridium* XIVa, and *Blautia*) (197). Similarly, Bacteroidales family members were found to be enriched in the gut microbiome of metastatic melanoma patients classified as non-responders to α-PD-1 therapy, while responders were found to exhibit greater microbial diversity in the gut and enrichment of members belonging to the Clostridiales order (198). It is possible that the differences reported in these clinical studies versus the study by Vetizou et al. (196) are due either to species-specific differences between mouse and man or to biased reconstitution of gut microbiota following fecal transplantation from humans to mice. However, it is worth noting that another clinical study comparing the baseline gut microbiota of responders versus non-responders to various checkpoint blockade regimens reported data from melanoma patients similar to that described by Vetizou et al.—that is, that enrichment of *Bacteroides* species correlated positively with patient response to therapy (199). In this most recent study, gut microbiome diversity was not significantly different in responders versus non-responders, but metagenomic shotgun sequencing analysis of pretreatment fecal samples identified enrichment of particular species in responding patients that was unique for each therapeutic regimen under study. When comparing responders versus non-responders to all checkpoint blockade regimens under study, both *Bacteroides caccae* and *Streptococcus parasanguinis* were enriched in the gut microbiomes of responders. When analyzing patients responding to ipilimumab/nivolumab combination therapy, Firmicutes phylum members (*Faecalibacterium prausnitzii* and *Holdemania filiformis*) and the Bacteroidetes phylum member *B. thetaiotaomicron* were enriched in responders. Finally, the Firmicutes phylum member *Dorea formicigenerans* was enriched in responders to therapy with pembrolizumab. Based on these collective data, it is clear that additional studies with larger cohorts of patients are necessary to resolve these early discrepant findings and determine how particular gut microbiota regulate both natural antitumor immune responses as well as responsiveness to various tumor immunotherapies. Additionally, as evidence is accumulating that the gut microbiome also influences immunometabolism (200) as well as the metabolism and antitumor activity of chemotherapeutic drugs (201), future studies are needed to investigate how particular microbial species and their metabolites regulate chemotherapy-driven ICD and the function of DC and other immune cell populations in the context of melanoma. Together, these insights will be important for the optimization of strategies to manipulate the gut microbiome in ways that enhance antitumor immune reactivity while also minimizing adverse events such as therapy-associated colitis (202).

### The Role of the Skin Microbiome in Immunity to Melanoma?

While a number of studies have been initiated to gain insights into the gut microbiome’s influence on the progression of melanoma and other cancers, little is currently known about how the skin microbiome might impact immunologic protection from either the development of primary melanomas or the recurrence of melanoma in the skin or surrounding/distant tissues. To date, only one study has compared the skin microbiome of cutaneous melanomas and benign melanocytic nevi (203). While the cutaneous microbial diversity of melanomas was found to be slightly lower than that of melanocytic nevi, these differences did not reach statistical significance, and no differences were found in the relative abundance of bacterial genera between patients from these groups. However, the limited sample size of this study (15 cutaneous melanoma cases versus 17 melanocytic nevi cases) precludes any strong conclusions that the skin microbiome has no impact on melanoma progression or anti-melanoma immunity in the skin. With regard to microbial influences on cutaneous immunity, others have reported associations between the skin microbiome and patient susceptibility to inflammatory skin conditions such as atopic dermatitis (204), and dysbiosis of the skin microflora has recently been linked to autoimmune vitiligo as well (205, 206). As vitiligo results from immune-mediated destruction of melanocytes, microbial species that influence this process may be of particular relevance to melanoma. In this light, a recent study comparing bacterial communities in lesional versus non-lesional skin of vitiligo patients revealed a decrease in microbial diversity in vitiliginous lesions, and intra-community network analyses showed that Actinobacterial species predominate the microbial interaction network of non-lesional skin, while members of the Firmicutes phylum exhibit the highest degree of interactions in lesional skin (205). Future studies will be necessary to determine the cause–effect relationship of these alterations in cutaneous microbial communities during cases of vitiligo and whether such alterations might also impact immune reactivity against melanoma cells. Answers to these questions and others that address how the cutaneous microbiota might influence the maturation/activation of Langerhans cells and other skin-resident DC populations may suggest microbial interventions that support the promotion of robust, DC-mediated anti-melanoma immune responses. Coupled with an improved understanding of the gut microbiome’s influence on DC-mediated immune responses against melanoma, these findings may identify appropriate dietary modifications, prebiotic/probiotic supplements, antibiotic regimens, and/or fecal transplantation strategies that can be implemented to support DC-based and other immune therapies for the treatment of melanoma.

## Conclusion and Future Directions

**Figure 4:**
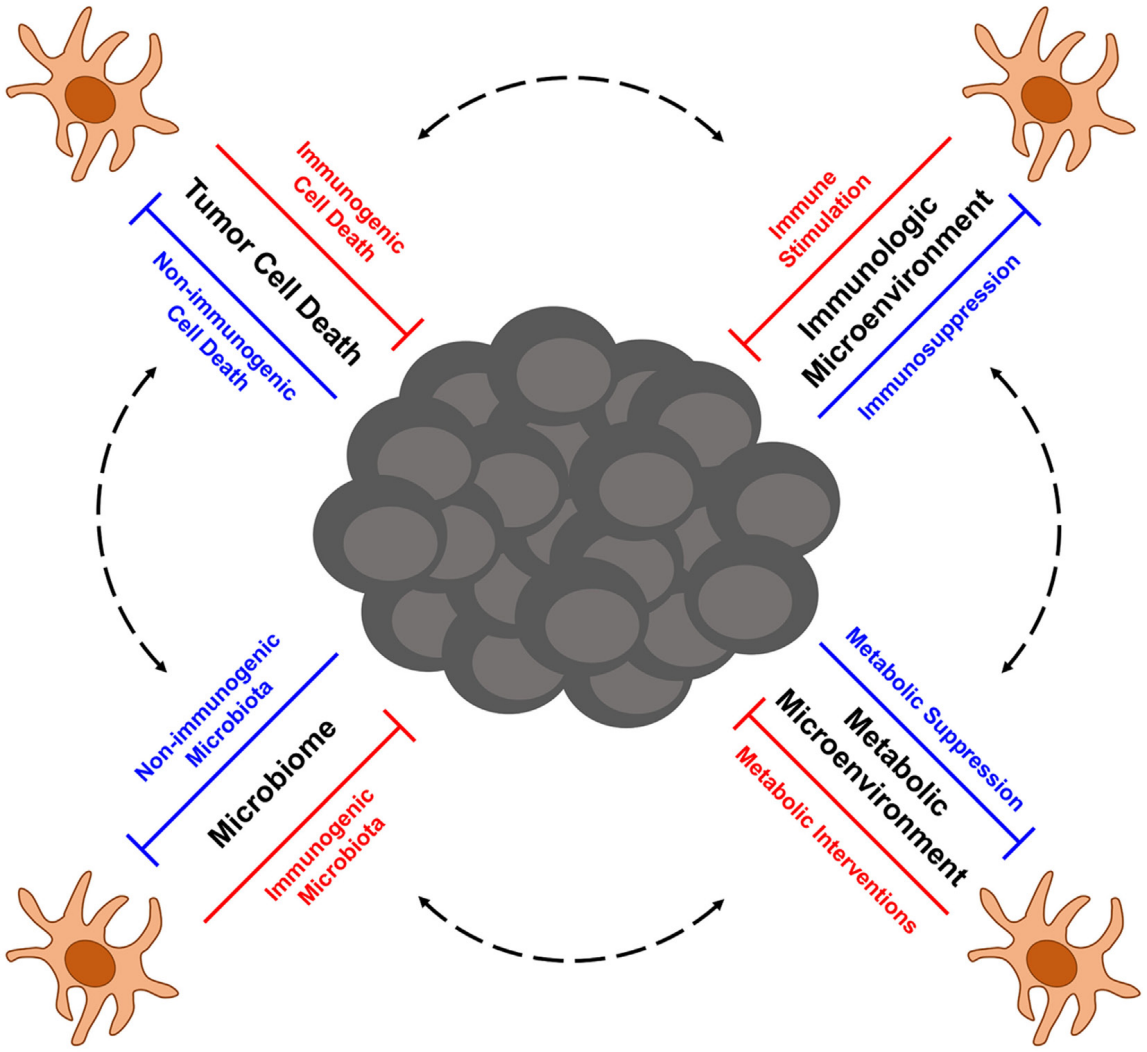
Multifactorial influences on the function of melanoma-associated dendritic cells (DC). A variety of complex factors contribute to the immunoregulation of DC in the context of melanoma. Elements that control the immunogenicity of tumor cell death, the balance of immunostimulatory versus immunosuppressive signals in the tumor microenvironment, metabolic influences on DC function, and the microbiome all interact to dictate the immune stimulatory capacity of melanoma-associated DC. Mechanistic insights into each of these layers of DC immune regulation provide opportunities for therapeutic interventions to enhance the immunogenicity and antitumor function of melanoma-associated DC as described in more detail in the text.

As highlighted throughout this review, DC function at the center of antitumor immunity and play major roles in determining immune activation versus tolerance against cancer. Regulation of immunity to melanoma by DC is controlled by a variety of intrinsic and extrinsic factors, and it is the collective interplay between these factors that ultimately shape the quality of DC-mediated antitumor immune responses (Figure 4). Advances in our understanding of the ways in which DC function is influenced by ICD, immunosuppressive networks within the tumor microenvironment, tumor-altered immunometabolism, and the microbiome have provided crucial insights into the immunoregulation of tumor-associated DC, and these insights have informed novel strategies for improving the immunogenicity of DC in the context of melanoma and other cancers. Some of these strategies have already reached patients and have improved the immunologic control of melanoma, and many others have shown great promise in murine models and in preclinical settings. It will therefore be exciting to follow the translation of these and related strategies for enhancing the immunostimulatory function of melanoma-associated DC into the clinic in the future. As we continue to build on these findings, the challenge going forward will be to dissect the complex interplay between the regulatory mechanisms discussed herein and discern how these diverse factors act in concert to control DC function. In this regard, in what ways does the microbiome impact the induction of ICD in melanoma cells? Can particular microbes provide metabolic support for DC by removing toxic byproducts from the tumor microenvironment, and how do microbe-derived metabolites themselves contribute to the metabolic milieu and its influence on DC in the tumor microenvironment? To what extent do immunosuppressive factors in the tumor microenvironment blunt DC function through regulation of metabolic pathways in these cells, and how might altering the balance of these factors impact the abundance and diversity of the microbiota and its contribution to tumor-associated DC function? Collectively, how do these factors influence the ability of DC to maintain the immune reactivity of T cells supported by checkpoint blockade therapy, and how might DC-based therapies best be utilized in combinatorial approaches to induce antitumor T cell responses in patients who have not mounted natural responses to melanoma and are therefore currently poor candidates for treatment by checkpoint blockade? These additional insights into DC immunoregulation in the context of melanoma, coupled with ongoing technological advances that enable fine-tuned manipulation of DC function, will arm scientists with the tools necessary to devise multifaceted approaches to overcome melanoma-imposed limitations on DC immunogenicity. Based on the advances that have already been seen in recent years, there is great optimism within the field that these novel approaches will significantly improve the antitumor efficacy and clinical outcome of DC-based immunotherapies for melanoma patients in the future.

## Author Contributions

KH was solely responsible for the conception and writing of this review article.

## Conflict of Interest Statement

The author declares that this review was written in the absence of any commercial or financial relationships that could be construed as a potential conflict of interest.
